# Opium use during pregnancy and infant size at birth: a cohort study

**DOI:** 10.1186/s12884-018-1994-8

**Published:** 2018-10-01

**Authors:** Siavash Maghsoudlou, Sven Cnattingius, Scott Montgomery, Mohsen Aarabi, Shahriar Semnani, Anna-Karin Wikström, Shahram Bahmanyar

**Affiliations:** 1 Clinical Epidemiology Unit, Department of Medicine, Karolinska Institute, Solna, Sweden; 2 Department of Obstetrics & Gynecology, McMaster University, 1280 Main Street West, room 3N52F, Hamilton, ON L8S 4K1 Canada; 3 Clinical Epidemiology and Biostatistics, School of Medical Sciences, Örebro University, Örebro, Sweden; 4 Department of Epidemiology and Public Health, University College London, London, UK; 5 Faculty of Medicine, Mazandaran University of Medical Sciences, Sari, Iran; 6 Faculty of Medicine, Golestan University of Medical Sciences, Gorgan, Iran; 7 Department of Clinical Sciences, Karolinska Institute, Danderyd Hospital, Stockholm, Sweden; 8 Clinical Epidemiology Unit & Centre for Pharmacoepidemiology, Department of Medicine, Karolinska Institute, Solna, Sweden

**Keywords:** Birth size, Iran, Opium, Pregnancy, Small for gestational age

## Abstract

**Background:**

The reported positive association between opiatic drug use during pregnancy and adverse pregnancy outcomes might be confounded by other factors related to high-risk behaviors, including the use of other harmful substances. In rural areas of Iran, opium use during pregnancy is relatively common among women who otherwise do not have a hazardous lifestyle, which reduces the risk of residual confounding and increasing the possibility to identify its effects. We aimed to examine the association of antenatal exposure to opium with risks of small for gestational age, short birth length, and small head circumference at birth.

**Method:**

In this cohort study in the rural area of the Golestan province, Iran, we randomly selected 920 women who were exposed to opium during pregnancy and 920 unexposed women during 2008–2010. Log-binomial regression was used to estimate risk ratios (RR) and 95% confidence intervals (CI) for the associations between prenatal exposure to opium and risks of small for gestational age, short birth length, and small head circumference at birth.

**Results:**

Compared with non-use of opium and tobacco during pregnancy, using opium only and dual use of opium and tobacco were associated with increased risks of small for gestational age at births (RR = 1.71; 95% CI 1.34–2.18 and RR = 1.62; 95% CI 1.13–2.30, respectively). Compared with non-use of opium and tobacco, exposure to only opium or dual use of opium and tobacco were also associated with more than doubled increased risks of short birth length, and small head circumference in term infants.

**Conclusion:**

Maternal opium use during pregnancy is associated with increased risks of giving birth to a small for gestational age infant, as well as a term infant with short birth length or small head circumference.

## Background

There has been an epidemic of opioid abuse worldwide, including North America, UK, Australia, and Asian countries [1, 2]. Opium (Poppy tears, Lachryma Papaveris, Theriac) has traditionally been used in Middle-eastern and south Asian countries, such as Iran, Afghanistan, Pakistan, India, and China, even during pregnancy [3]. Opium obtained from the poppy plant, Papaver somniferous, contains morphine (10% of opium), Codeine (0.5%), Thebaine (0.2%), Papaverine (1%), and Noscapine (6%) [4].

Use of opioids during pregnancy, including heroin and methadone, has been associated with adverse pregnancy outcomes, such as preterm delivery, low birth weight, SGA, and neonatal complications [5]. However, these studies were performed on drug addicts in high-income countries, and the reported effect of the opioid use on pregnancy outcomes might be confounded by social factors, lifestyle and other factors related to high-risk behaviors, including the use of other harmful substances [6]. Thus, differentiating the effects of opioid use from the effects of other risk factors has been difficult [7].

In Golestan province, northern Iran, some women use (mainly smoke) opium [8]. In this area, opium-using women are mostly homemakers, who otherwise do not have a hazardous lifestyle – such as homelessness and criminal behavior – which reduces the risk of residual confounding [9, 10]. In a cohort study from north-east Iran, we used prospectively collected information before and during pregnancy to examine the association of antenatal exposure to opium with anthropometric characteristics of the newborn infants, including weight, length, and head circumference at birth.

## Methods

A detailed description of the study population and method appears elsewhere [11]. Briefly, we performed a cohort study in rural areas of Golestan province, in northeast Iran, between 2008 and 2010. The province has approximately 1, 700, 000 inhabitants. Around 50% are living in rural areas, where there are approximately 280, 000 women in reproductive age and 17, 000 births annually. Based on the national public health care system in Iran, each village has at least one rural health house, which is part of the primary health care organization and works under the authority of more specialized health care centers. The health houses are responsible for providing care and recording of health-related data, including medical information before and during pregnancy. Medical information including maternal drug use, cigarette smoking, alcohol use, and other high-risk behaviors are recorded both before and during pregnancy [9]. Opium is the most common drug in Golestan province and is usually smoked [12].

The exposed group included women with a self-reported history of using any of a variety of opiates during the index pregnancy. The unexposed group included women who did not use opium before or during pregnancy. We used the stratified random sampling method to select exposed and unexposed groups. Each of the 13 regions of the province was determined as a block, and the sample size of each block was estimated based on the population growth rate of the region. There were 30, 868 pregnancies during the study period. Exposed pregnancies were randomly selected, using computer-created random digits and the same number of women unexposed to opium during pregnancy were frequency-matched to the exposed women by the residential area (village). Information on pregnancies was abstracted from medical records and computerized. As a quality control, some 10% of the data was collected a second time. We recollected the data if there were more than 5% mismatches.

We selected a total of 920 opium users during pregnancy (“exposed” women) and 920 non-opium users (“unexposed” women). Thereafter, we excluded women with multiple births, miscarriages, and stillbirths, also women who gave up using opium in early pregnancy, used alcohol during pregnancy, and tobacco smokers among the unexposed group (15 unexposed and 33 exposed mothers). Finally, our study included 887 opium users (of whom 210 also smoked tobacco, and 677 only used opium) and 905 non-opium users with live singleton births.

Information on socioeconomic circumstances was based on the husband’s profession (categorized as an unskilled manual worker, skilled manual worker, self-employed, farmer, other occupations, and unemployed). Agricultural production is the main economic sector in Golestan province. Gestational age was based on the time interval between the date of the first day of the last menstrual period and the date of delivery. Preterm delivery was defined as a delivery before 37 completed gestational weeks. Small for gestational age (SGA) was defined as a birth weight below the 10th percentile for gestational age, based on a sex-specific global reference for birth weight [13]. The 5th percentile of birth length (crown-heel length) and head (Occipito-Frontal) circumference among unexposed and term pregnancies were considered as cut-offs for short birth length (shorter than 47 cm) and small head circumference (smaller than 33 cm), respectively.

We used univariate and multivariate log-binomial regression models to estimate risk ratios (RRs) and 95% confidence intervals (CIs) for the associations between opium use during pregnancy and SGA, short birth length, and small head circumference. The models were adjusted for potential confounding factors, including the region of residence, maternal age, parity, height, body mass index, husband’s occupation, and infant sex. Due to scarce data on preterm infants, we could not construct gestational-age specific curves for birth length and head circumference. We restricted the analyses of birth length and head circumference to term births (≥ 37 weeks), where we additionally also adjusted for gestational age (in weeks). Stratified analyses were performed to investigate the risks of SGA, short birth length, and small head circumference among opium-using mothers who smoked or did not smoke tobacco during pregnancy.

We used multiple imputation method with five imputations approach was used to provide data where there were missing values for maternal age (6 among mothers unexposed to opium and 5 among exposed mothers), infant sex (15 among mothers unexposed to opium and 10 among exposed mothers), husband’s occupation (4 among unexposed and 9 among exposed), and mother’s height (39 among unexposed and 17 among exposed). SAS software version 9.4 was used for all analyses.

## Results

Table 1 shows maternal and births characteristics in unexposed, all exposed, exposed to opium only and exposed to both opium and tobacco. Among unexposed women, 28% were 30 years or older, and 59% were parous. For women exposed to opium corresponding figures were 44% and 78%, respectively. There were no differences between the cohorts regarding other maternal characteristics. Among infants prenatally unexposed and exposed to opium, 6.5% and 13.0% were shorter than 47 cm at birth, respectively, while corresponding percentages for small head circumference (< 33 cm) were 6.2% and 13.4%, respectively (Table 1).

**Table 1:** Characteristics of study subjects

	Unexposed ^a^	Opium user		
		All	Opium user only	Tobacco and Opium user
	*N* (%)	*N* (%)	*N* (%)	*N* (%)
Maternal age (Years)				
≤ 19	134 (14.8)	49 (5.5)	40 (5.9)	9 (4.3)
20–24	250 (27.6)	161 (18.2)	133 (19.6)	28 (13.3)
25–29	260 (28.7)	278 (31.3)	210 (31.0)	68 (32.4)
30–34	187 (20.7)	251 (28.3)	181 (26.7)	70 (33.3)
≥ 35	68 (7.5)	143 (16.1)	108 (16.0)	35 (16.7)
Missing	6 (0.7)	5 (0.6)	5 (0.7)	0 (0)
Parity				
Nulliparous	372 (41.1)	199 (22.4)	155 (22.9)	44 (21.0)
Parous	533 (58.9)	688 (77.6)	522 (77.1)	169 (79.0)
Maternal height (cm)				
≤ 149	68 (7.5)	66 (7.4)	47 (6.9)	19 (9.0)
150–155	198 (21.9)	193 (21.8)	152 (22.5)	41 (19.5)
156–160	380 (42.0)	399 (45.0)	295 (43.6)	104 (49.5)
161–164	120 (13.3)	109 (12.3)	84 (12.4)	25 (11.9)
≥ 165	100 (11.0)	103 (11.6)	86 (12.7)	17 (8.1)
Missing	39 (4.3)	18 (2.0)	13 (1.9)	4 (1.9)
Maternal body mass index				
< 18.5	63 (7.0)	52 (5.9)	34 (5.0)	18 (8.6)
18.5 to < 25	439 (48.5)	474 (53.4)	356 (52.6)	118 (56.2)
25 to < 30	245 (27.1)	231 (26.0)	184 (27.2)	47 (22.4)
30 to < 35	91 (10.1)	91 (10.3)	72 (10.6)	19 (9.0)
≥ 35	28 (3.1)	21 (2.4)	18 (2.7)	3 (1.4)
Missing	39 (4.3)	17 (1.9)	13 (1.9)	5 (2.4)
Husband’s occupation				
Unemployed	25 (2.8)	56 (6.3)	37 (5.5)	19 (9.0)
Non skill worker	400 (44.2)	419 (47.2)	312 (46.1)	107 (51.0)
Skill worker	91 (10.1)	70 (7.9)	58 (8.6)	12 (5.7)
Self-employed	111 (12.3)	101 (11.4)	89 (13.1)	12 (5.7)
Farmer	179 (19.8)	163 (18.4)	123 (18.2)	40 (19.0)
Other	95 (10.5)	69 (7.8)	52 (7.7)	17 (78.1)
Missing	4 (0.4)	9 (1.0)	6 (0.9)	3 (1.4)
Unemployed	25 (2.8)	56 (6.3)	37 (5.5)	19 (9.0)
Preterm delivery				
Term	850 (93.9)	798 (90.0)	616 (91.0)	182 (86.7)
Preterm	55 (6.1)	89 (10.0)	61 (9.0)	28 (13.3)
Infant sex				
Female	447 (49.4)	436 (49.2)	337 (49.8)	99 (47.1)
Male	443 (49.0)	441 (49.7)	331 (48.9)	110 (52.4)
Missing	15 (1.7)	10 (1.1)	9 (1.3)	1 (0.5)
Birth weight (gr)				
≤ 2500	43 (4.8)	99 (11.2)	75 (11.1)	24 (11.4)
2500–2999	139 (15.4)	179 (20.2)	134 (19.8)	45 (21.4)
3000–3299	205 (22.7)	203 (22.9)	143 (21.1)	60 (28.6)
3300–3599	269 (29.7)	193 (21.8)	154 (22.7)	39 (18.6)
≥ 3600	234 (25.9)	203 (22.9)	162 (23.9)	41 (19.5)
Missing	15 (1.7)	10 (1.1)	9 (1.3)	1 (0.5)
Birth length (cm)				
≤ 46	59 (6.5)	115 (13.0)	87 (12.9)	28 (13.3)
47–48	149 (16.5)	138 (15.6)	103 (15.2)	35 (16.7)
49–51	535 (59.1)	480 (54.1)	359 (53.0)	121 (57.6)
≥ 52	147 (16.2)	144 (16.2)	119 (17.6)	25 (11.9)
Missing	15 (1.7)	10 (1.1)	9 (1.3)	1 (0.5)
Head circumference (cm)				
≤ 32	56 (6.2)	119 (13.4)	88 (13.0)	31 (14.8)
33–34	242 (16.7)	337 (38.0)	252 (37.2)	85 (40..5)
35–36	476 (52.6)	362 (40.8)	281 (41.5)	81 (38.6)
≥ 37	116 (12.8)	59 (6.7)	47 (6.9)	12 (5.7)
Missing	15 (1.7)	10 (1.1)	9 (1.3)	1 (0.5)

^a^Unexposed includes mothers who are non-users of both opium and tobacco

Compared with non-use of opium and tobacco, use of opium during pregnancy was associated with a 69% increased risk of birth of an SGA infant after adjusting for potential confounders. The risk of SGA was slightly higher in offspring of mothers who only used opium (RR = 1.71 and 95% CI; 1.34–2.18) than in offspring of mothers who both used opium and smoked tobacco during pregnancy (RR = 1.62 and 95% CI; 1.13–2.30). Meanwhile, when we restricted the analysis into the term pregnancies, the risk of SGA was almost identical in offspring of mothers who only used opium (RR = 1.58 and 95% CI; 1.22–2. 04), and offspring of dual user mothers (RR = 1.58 and 95% CI; 1.09–2.29) (Table 2).

**Table 2:** Risk ratios (RR) and 95% confidence intervals (CI) for the associations between opium and tobacco use during pregnancy and risk of small for gestational age at birth

	Pregnancies	Small for gestational age ^a^	Crude	Adjusted ^b^
	*N*	*N* (%)	RR (95% CI)	RR (95% CI)
Opium				
Unexposed	905	99 (10.9)	Reference	
Exposed	887	154 (17.4)	1.59 (1.26–1.99)	1.69 (1.34–2.13)
Tobacco and opium				
Unexposed	905	99 (10.9)	Reference	
Exposed to opium only	677	115 (17.0)	1.59 (1.22–2.06)	1.71 (1.34–2.18)
Exposed to tobacco and opium	210	39 (18.6)	1.58 (1.10–2.28)	1.62 (1.13–2.30)
Restricted to term pregnancies				
Opium				
Unexposed	850	93 (10.9)	Reference	
Exposed	798	132 (16.5)	1.46 (1.15–1.86)	1.58 (1.23–2.02)
Tobacco and opium				
Unexposed	850	93 (10.9)	Reference	
Exposed to opium only	616	97 (15.7)	1.43 (1.11–1.85)	1.58 (1.22–2.04)
Exposed to tobacco and opium	182	35 (19.2)	1.57 (1.10–2.24)	1.58 (1.09–2.29)

^a^Small for gestational age was defined as a birth weight below the 10th percentile for appropriate gestational age, based on a sex-specific global reference for birth weight [13]

^b^Adjusted for: maternal age, height, BMI, parity, husband’s occupation, and residential place

Compared with prenatally unexposed term infants, term infants who were prenatally exposed to opium had a more than doubled risk of short birth length (< 47 cm). The risk of short birth length was essentially similar between those exposed to only opium (RR = 2.00 and 95% CI; 1.37–2.92) and those exposed to both opium and tobacco during pregnancy (OR = 2.40 and 95% CI; 1.20–3.48) (Table 3).

**Table 3:** Risk ratios (RR) and 95% confidence intervals (CI) for the associations between opium and tobacco use during pregnancy and risk of short birth length at term birth

	Pregnancies	Short birth length ^a^	Crude	Adjusted ^b^
	*N*	*N* (%)	RR (95% CI)	RR (95% CI)
Opium				
Unexposed	850	50 (5.9)	Reference	
Exposed	798	92 (11.5)	1.95 (1.38–2.75)	2.01 (1.40–2.89)
Tobacco and opium				
Unexposed	850	50 (5.9)	Reference	
Exposed to opium only	616	70 (11.4)	1.93 (1.36–2.73)	2.00 (1.37–2.92)
Exposed to tobacco and opium	182	22 (12.2)	2.03 (1.26–3.27)	2.04 (1.20–3.48)

^a^Birth length less than 47 cm (below the 5th percentile of birth length of unexposed and term live birth)

^b^Adjusted for: maternal age, height, BMI, parity, weeks gestational age, infant sex, husband’s occupation, and residential place

Compared with prenatally unexposed term infants, term infants who were prenatally exposed to opium had a 2.2-fold risk of the small head circumference at birth. The risk of a small head circumference was similar in infants prenatally exposed to only opium and in infants exposed to both opium and tobacco (Table 4).

**Table 4:** Risk ratios (RR) and 95% confidence intervals (CI) for the associations between opium and tobacco use during pregnancy and risk of small head circumference at term birth

	Pregnancies	Small head circumference ^a^	Crude	Adjusted ^b^
	*N*	*N* (%)	RR (95% CI)	RR (95% CI)
Opium				
Unexposed	850	45 (5.3)	Reference	
Exposed	798	99 (12.4)	2.33 (1.64–3.32)	2.24 (1.55–3.24)
Tobacco and opium				
Unexposed	850	45 (5.3)	Reference	
Exposed to opium only	616	76 (12.3)	2.33 (1.61–3.36)	2.24 (1.53–3.29)
Exposed to tobacco and opium	182	23 (12.6)	2.36 (1.43–3.90)	2.23 (1.31–3.79)

^a^Small head circumference less than 33 cm (below the 5th percentile of head circumference of unexposed and term live birth)

^b^Adjusted for: maternal age, height, BMI, parity, husband’s occupation, infant sex, weeks gestational age, and residential place

## Discussion

This cohort study revealed an increased risk of SGA at birth among offspring of mothers who used opium during pregnancy. This study showed that prenatal opium exposure also increased the risks of a short birth length and a small head circumference in infants born at term.

Our findings are consistent and extend results of previous studies showing that infants of mothers using heroin, methadone, or other opiates during pregnancy intend to have a higher risk of low birth weight [14, 15]. Meta-analyses have shown that use of any kind of opiates during pregnancy is associated with a three-fold increased risk of perinatal mortality and a four-fold increased risk of low birth weight [16]. We found that infants prenatally exposed to opium were at increased risk of being born SGA, but the risk of SGA was similar among infants only prenatally exposed to opium and those exposed to both opium and tobacco. As maternal smoking is causally associated with fetal growth restriction, we expected a higher risk among infants exposed to both opium and tobacco [17]. We cannot explain the lack of additive effect of opium and tobacco on SGA. Residual confounding may be one possibility, but the lack of observed effect may also be due to chance [17].

Our study demonstrated that term infants who were exposed to opium had shorter birth length after adjusting for gestational age, sex and other potential confounders. An increased risk of a short birth length was also observed in studies investigating maternal heroin or methadone use during pregnancy [18]. Liu et al. reported that infants who were prenatally exposed to opium or methadone had shorter birth length compared with unexposed infants [15]. An ultrasonic investigation during pregnancy showed that fetuses exposed to opiates had shorter femur and humerus lengths compared with unexposed fetus [19].

In this study, we also found that opium exposure during pregnancy is associated with a reduced head circumference, which is consistent with results of previous studies on maternal heroin or methadone use during pregnancy [15]. Hunt et al. reported that neonates prenatally exposed to opiates on average had smaller head circumference at birth. However, the study included both term and preterm infants, and the exposed group were born at a lower gestational age than the unexposed group [20]. A Swiss study also documented a four-fold increased risk of the small head circumference at birth (smaller than the third percentile) among neonates of methadone-addicted mothers [21]. Previous studies reported children of mothers using opiate during pregnancy had higher risks of intentional disorders, impaired motors, and cognitive functions, at school ages which could be due to impaired neurodevelopment [20, 22]. The long-term effects of opium exposure in utero on psycho-behavioral dysfunctions in offspring have also been demonstrated in other studies [23].

Several explanations have been proposed for the associations of opiates exposures during pregnancy with risks of fetal brain development and growth restrictions. Using opiate during pregnancy is associated with risks of preeclampsia, premature labor, premature rupture of membranes, placental abruption, placental insufficiency and intrauterine death [24]. Maternal narcotic use induces hypoxia and asphyxia in the fetus, which is a consequence of fluctuating overdose-withdrawal cycles during pregnancy [25]. Moreover, animal studies showed a direct effect of opiates on the neural elements of the fetus [26]. It has also been suggested that factors such as parental psychopathology, maternal multiple drug abuse, poor prenatal care, dietary restriction, and disadvantaged socioeconomic circumstances could mediate associations between opiates exposures during pregnancy and risks of fetal brain development and growth restriction [27]. However, Pinto et al. reported that despite comprehensive antenatal care, drug-using women are at higher risk of giving birth to a low birth weight infant [5].

Opium has traditionally been used in southern Asia and middle-eastern countries such as Iran as a sedative and for pleasure. It has been shown that opium users have less psychiatric comorbidity, unemployment, homelessness, and criminal behavior compared with users of other narcotic drugs [10, 28]. In Iran, the socioeconomic profile of opium users was almost identical with the socioeconomic circumstance of the general population [8]. In rural areas of Golestan province, where most of them are housewives, their pregnancies are planned, and approximately 97% of all pregnant women have access to primary health service funded by the state [29].

Strengths of this study include the cohort design, using prospectively collected information from antenatal visits before and during pregnancy. The population was relatively homogeneous, and opium-exposed mothers had otherwise had a normal lifestyle and an acceptable level of care during pregnancy [10]. We excluded a small proportion of women who were exposed to alcohol and selected non-users of opium and tobacco as the reference group to increase internal validity. Potential limitations include a lack of detailed information on mothers’ socioeconomic circumstances. However, we controlled for husband’s occupation and region of residence. Information on exposure to opium and smoking during pregnancy was based on self-reports during routine visits. There was no information on the frequency or dose of opium to estimate dose-response associations. If mothers did not report their drug use, this may have pushed our estimates toward the null. However, the reliability of self-reported opium use in rural Golestan has been reported as high [30]. Gestational age was estimated based on information on the first day of the last maternal menstrual period, which can be subject to error [31]. Finally, as the study was based on data on pregnancies in the rural area of Golestan province, there may be concern regarding the generalizability of the results.

## Conclusions

Maternal opium use during pregnancy is associated with giving birth to small for gestational age infants, and with short birth length and small head circumference in term infants. It is unlikely that the observed associations are confounded by other factors related to high-risk behavior, such as the use of other harmful substances. Preventative intervention studies should be conducted in regions where opium use is common among young women.

## Abbreviations

BMI: Body Mass Index
CIs: Confidence Intervals
cm: centimeter
RRs: Risk Ratios
SGA: Small for Gestational Age
